# A Membrane-Based Process for the Recovery of Glycyrrhizin and Phenolic Compounds from Licorice Wastewaters

**DOI:** 10.3390/molecules24122279

**Published:** 2019-06-19

**Authors:** Carmela Conidi, Lidia Fucà, Enrico Drioli, Alfredo Cassano

**Affiliations:** Institute on Membrane Technology, ITM-CNR, c/o University of Calabria, via P. Bucci, 17/C, I-87036 Rende, Cosenza, Italy; lidiafuca@gmail.com (L.F.); e.drioli@itm.cnr.it (E.D.)

**Keywords:** licorice wastewaters, glycyrrhizin, bioactive compounds, ultrafiltration, nanofiltration

## Abstract

In this work, the use of polymeric ultrafiltration and nanofiltration membranes was investigated in order to recover glycyrrhizin and phenolic compounds from licorice wastewaters. Filtration experiments were performed on a laboratory scale using four polyamide thin-film composite membranes (GK, GH, GE, and DK, from GE Osmonics) with different molecular weight cut-offs (from 150 to 3500 Da). The permeate flux and retention values of glycyrrhizin, the total polyphenols, the caffeic acid, the total carbohydrate, and the total antioxidant activity as a function of the transmembrane pressure (TMP) and weight reduction factor (WRF) were evaluated. In selected operating conditions, the membrane productivity decreased in the order of GK > DK > GH > GE, with a similar trend to that of water permeability. Glycyrrhizin was totally rejected by selected membranes, independently of TMP and WRF. For the other antioxidant compounds, the retention values increased by increasing both of the parameters. According to the experimental results, a combination of membranes in a sequential design was proposed as a viable approach to produce concentrated fractions enriched in bioactive compounds and purified water from licorice wastewater.

## 1. Introduction

Licorice (*Glycyrrhiza glabra* L.) is a ligneous perennial shrub of the fabaceae family, growing mainly in central Asia, the Middle East, and south eastern Europe. The dried roots of licorice are widely used as a food flavoring agent and for various medicinal purposes since ancient times [1]. As such, preparations of licorice root are recognized for their significant antioxidant, anti-inflammatory, anti-microbial, anti-viral, and anti-carcinogenic activity [2], as well as for their hepatoprotective effects [3]. These activities are linked to a characteristic chemical composition, which includes several bioactive compounds, including glycyrrhizin (~16%), different sugars (up to 18%), flavonoids, saponoids, sterols, starches, amino acids, gums, and essential oils [4]. The major active component of licorice is glycyrrhizin or glycyrrhizic acid, a triterpenoid glycoside compound with a high-intensive sweetness (about 50 times greater than sucrose) used extensively throughout the world as a functional additive in food, beverages, cosmetics, and tobacco industries. 

The conventional manufacturing method of licorice is based on decoction, as follows: The roots are first harvested and dried, after which they are cleaned to remove physical impurities, such soil and the like. Licorice roots are then ground into powder and boiled, sometimes under pressure. The extract is then filtered or decanted and concentrated by evaporation, in order to obtain a product with the desired moisture content. During licorice processing, a characteristic wastewater stream, yellow to brown in color, is generated from the cleaning of the pipes and extraction equipment. This effluent is characterized by a high chemical oxygen demand (COD) (up to 8000 mg/L), which is prohibited from being directly discharged into the water or onto land. In addition, the presence of phenolic compounds makes licorice wastewaters phytotoxic, inhibiting bacterial activity. Until now, few studies have reported about the pollution control of these wastewaters. A combination of physicochemical and biological processes, including adsorption powdered activated carbon (PAC), flocculation, upflow anaerobic sludge blanket (UASB) reactor, and contact oxidation, have been reported by Guo et al. [5] for the treatment of licorice wastewaters. Recently, Ramaswami et al. [6] reported a significant reduction of the COD (up to 80%) in the pretreatment of licorice processing wastewater by the activated sludge process. The removal of residual organics and color from the treated effluent can be implemented by activated carbon adsorption, although at a high carbon dosage.

As for other plant-derived wastes, licorice wastewaters represent a major source of bioactive compounds with a great potential for recovery or conversion to valuable products within the logic of the biorefinery concept, leading to significant advantages in terms of the full utilization of feedstocks, minimization of waste generation during processing, and diversification of the revenues through the formulation of products for different market areas [7].

The purification of glycyrrhizic acid from the natural extract of licorice is still a challenge because of the complex composition of the extract and interferences with other compounds, especially flavonoids. The extraction of glycyrrhizic acid from licorice by the conventional liquid–liquid extraction technique is characterized by several disadvantages, such as high extraction times and temperatures, large solvent requirements, and low yields. Macroporous resins [8], ultrasound-estraction [9], microwave-assisted extraction [10], supercritical CO_2_ extraction [11], high-speed [12], and pH-zone-refining counter-current chromatography [13] have also been adopted. However, most of these procedures involve a number of steps and large amounts of solvent/chemicals; additional disadvantages include poor recovery (from 20% to 30%) and high process costs.

The concentration and selective fractionation of bioactive compounds from aqueous and alcoholic processing streams of agro-food production by pressure-driven membrane processes, including ultrafiltration (UF) and nanofiltration (NF), have been extensively studied in the last years [14,15,16]. These processes offer several advantages when compared with conventional separation methodologies. Indeed, they can be operated in mild operating conditions without changing the physical state of the solvent, thus allowing for preserving the functional properties of the compounds of interest; easy automation and scale-up, shorter processing time, and lower labor and energy costs are additional advantages.

To date, few literature data have reported the use of membrane processes for the extraction and purification of bioactive compounds from licorice root extracts. Madaeni et al. [17] evaluated the separation feasibility of liquiritin from glycyrrhizic acid in licorice root extracts by using a polymeric NF membrane (NRT-7450 from Hydranautics) with a molecular weight cut-off (MWCO) in the range of 600–700 Da. A process for separating glycyrrhizic acid from licorice root extract based on the use of UF membranes was also patented by Gorgol et al. [18].

To the best of our knowledge, no literature is readily available on the performance of membrane-based processes in separating and concentrating bioactive compounds from licorice wastewater. For this reason, the main contribution of this work focuses on the evaluation of UF and NF membranes’ capability to separate and concentrate bioactive compounds from licorice wastewaters, in order to develop natural products suitable for pharmaceutical, cosmeceutical, and nutraceutical applications.

Licorice wastewaters were submitted to a preliminary UF step devoted to the removal of macromolecular compounds and suspended solids. The clarified solution was then fractionated by using tight UF and NF polymeric membranes with different MWCOs. The performance of the selected membranes was evaluated in terms of the productivity and separation capability towards biologically active compounds, including glicyrrhizic acid and caffeic acid.

## 2. Results and Discussion

### 2.1. Clarification of Licorice Wastewater 

**Figure 1:**
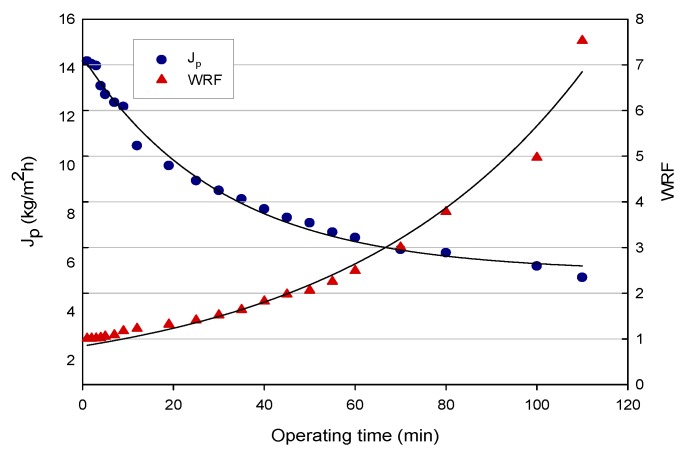
Clarification of licorice root extracts by ultrafiltration (UF). Time course of permeate flux and weight reduction factor (WRF; T 24 ± 1 °C; TMP 0.26 bar; Q_f_, 516 L/h).

In Figure 1, the time evolution of the permeate flux and WRF in the clarification of licorice wastewater in selected operating conditions is reported. The initial permeate flux of about 14 kg/m^2^ h decreased gradually down to 4.8 kg/m^2^ h when a final WRF value of 7.5 was reached. As a general trend, the permeate flux decreased by 42% in the first stage, from 0 to 50 min, followed by a smaller flux decline without reaching a steady-state value. This behavior can be attributed to different phenomena, which include concentration polarization, membrane fouling, and increased concentration of solutes in the retentate stream. Indeed, as the feed concentration increases, the concentration polarization becomes more severe. More solutes are convected towards the membrane surface, resulting in a thicker cake layer. This increases the resistance against the solvent flux, and the permeate flux declines [19].

The initial water permeability of the UF membrane of about 193 kg/m^2^ h bar decreased by about 57.1% after the treatment with the licorice wastewater; after the chemical cleaning with an alkaline solution, they were allowed to recover about 80% of the initial water permeability of the membrane. These results indicate that the internal fouling effects were more effective than the cake layer effects during the clarification of licorice wastewater. 

The general composition of licorice wastewater, before and after the clarification process, is reported in Table 1. The UF treatment allowed for removing all of the suspended solids with the production of a clear solution.

As previously reported, glycyrrhizic acid is the main biologically active ingredient of the licorice root. The amount of glycyrrhizic acid obtained from different species is highly variable, depending on different parameters, such as the plant age, season harvest, and climate conditions. Typically, it ranges from 0.2 to 83.7 mg/g [20]. 

The glycyrrhizic acid content of the UF feed is about 224 mg/L. As expected, glycyrrhizic acid was recovered in the permeate stream, on the basis of its molecular weight (822.06 g/mol) and the MWCO of the UF membrane (50 kDa). Similarly, most of the caffeic acid and total polyphenols were recovered in the clarified fraction, because of the low rejection of the membrane towards these components (in the range 4%–10%). The content of the total polyphenols in the feed solution resulted in similar results to that of the extracts generated at 200 °C for 30 min through the subcritical water extraction of licorice roots [21]. No significant difference was observed in the TAA of the UF permeate in comparison with the UF feed, as the total polyphenols, caffeic acid, and glycyrrhizic acid contribute to the antioxidant activity of licorice root extracts [4]. Adversely, a decrease in the content of the total carbohydrates (of about 15%) in the UF permeate was measured. This behavior could be attributed to the interaction of carbohydrates with suspended solids.

### 2.2. UF and NF of Clarified Licorice Wastewaters: Flux Evaluation

**Figure 2:**
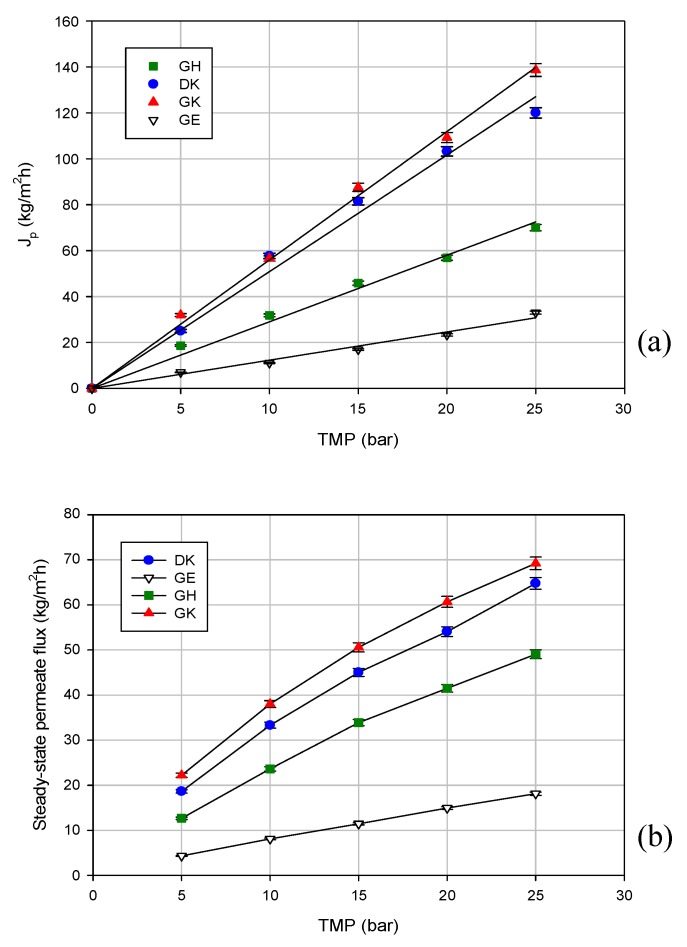
Permeate flux variation with transmembrane pressure (TMP) for selected membranes using the following: (**a**) deionized water; (**b**) clarified licorice wastewater (T 23 ± 1 °C; Q_f_ 480 L/h).

Preliminary UF and NF experiments were performed according to the full recycle mode, in order to assess the influence of the TMP on the permeate flux and the selectivity towards the target compounds. Prior to the general filtration experiments of the wastewater, filtration experiments with pure water were carried out in order measure the evolution of water permeate flux with the variation of TMP. The water flux increased linearly with the TMP in the range of the pressure investigated (5–25 bar; Figure 2a). For the UF membranes, the water flux increased by increasing the MWCO, as follows: specifically, the highest permeability was observed for the GK membrane of 3500 Da. It is well known that the water permeability is an inherent characteristic related to the composition, morphology, pore dimension, and hydrophobicity/hydrophilicity (wettability) of the membranes, and as such, it is not indicative of process flux [22]. In the present case, as also reported in Table 4, the DK membrane exhibited a lower contact angle than those reported for the UF membranes (indicating a higher wettability). This factor can explain the higher water permeability of the DK membrane in comparison with the GH and GE membranes, despite its lower MWCO. A similar behavior was observed in the treatment of the clarified extract.

Figure 2b shows the effect of TMP on the steady-state permeate flux for the selected membranes: the GK membrane, with the highest MWCO, exhibited the highest steady-state permeate fluxes (permeate flux was improved from 22 to 69 kg/m^2^ h by increasing the TMP from 5 to 25 bar), followed by the DK membrane with the lowest MWCO (permeate flux from 19 to 64 kg/m^2^ h in the same range of TMP). The lowest permeate flux values were recorded for the GE membrane (MWCO of 1000 Da). A similar behavior was observed for the treatment of aqueous extracts from distilled fermented grape pomace using UF and NF membranes [23]. Among the different tested membranes, the Nanomax 95 (from Millipore, Millipore, Bedford, MA), with the lowest MWCO (250 Da) exhibited the highest flux. Cissé et al. [24] evaluated the performance of different UF and NF membranes in the concentration of anthocyanins in acai juice and roselle extracts, obtaining similar results. Among the NF membranes investigated, a polyamide NF membrane with a MWCO of 250 Da (NF270, from Dow Filmtec, Midland, MI, USA) showed higher permeate fluxes when compared with the polyethersulphone NF membranes (NP010 and NP030 from Mycrodin-Nadir, Wiesbaden, Germany) with a higher MWCO. These results confirm that the permeate flux is affected by the membrane material and structure, as well as by the interaction between the solute and membrane.

According to the data reported in Figure 2b, the permeate flux increases in the range of the investigated operating pressures, and a TMP limit is not reached at all for all of the selected membranes independently of the MWCO. These results confirm less fouling, because the steady-state flux shows a closer agreement with the non-fouled membrane (i.e., flux linear with pressure). This is consistent with other studies performed with different aqueous extracts. A linear trend between the permeate flux and TMP was reported by Madaeni et al. [17] in the processing of licorice root extract through a polyamide NF membrane (NRT-7450, from Hydranautics, Oceanside, CA, USA) with a MWCO in the range of 600–700 Da. Similarly, Cassano et al. [25] observed a linear increase of permeate flux with pressure in the treatment of ultrafiltered artichoke brine using a DK membrane. As reported by the authors, this phenomenon can be attributed to the preliminary UF treatment, which reduces the fouling layer and the specific resistance of the membrane. 

**Figure 3:**
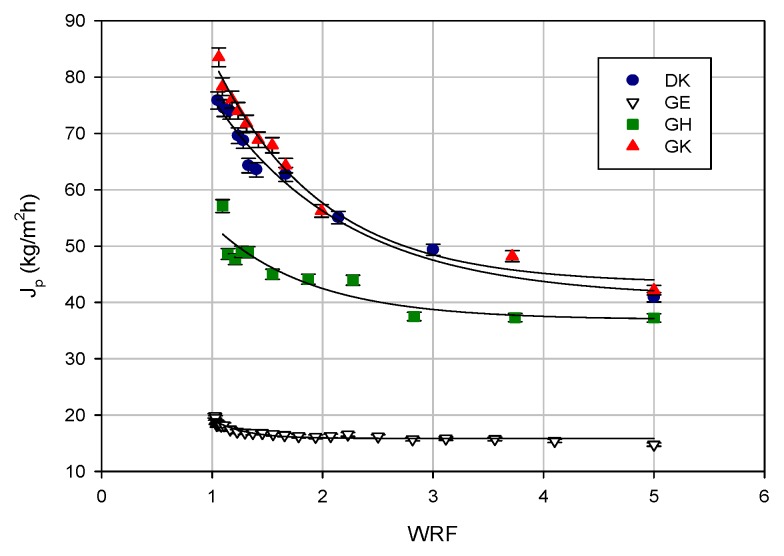
Treatment of clarified licorice wastewater by UF and nanofiltration (NF) membranes. Permeate flux as a function of the weight reduction factor (WRF; TMP 25 bar; Q_f_ 480 L/h; T 23 ± 1 °C).

Concentration experiments were performed at a TMP of 25 bar, an operating temperature of 25 °C, and a feed flowrate of 480 L/h, until reaching a WRF of 5. The evolution of the permeate flux with the WRF for the selected membranes is represented in Figure 3. For most of the selected membranes, the permeate flux decreased sharply because of the rapid build-up of concentration of polarization at the membrane–liquid interface, followed by a thicker and denser solute layer, which reduces the permeate flux until it reaches a steady-state condition. A similar progressive permeate flux decline was observed by Sohrabi et al. [26], in the treatment of a licorice aqueous solution with a NF membrane (BDX NF-90 from Fairway, Hang Zhou, China), at a pH of 5 and an operating pressure of 12 bar. 

The results confirmed the highest permeate fluxes for the GK membranes, followed by the DK membrane. In particular, for the GK membranes, the initial permeate flux of 83.5 kg/m^2^ h decreased by about 50% at the final WRF of 5; for the DK membrane, the initial permeate flux was about 76 kg/m^2^ h. Both of the membranes showed a permeate flux higher than 40 kg/m^2^ h in the correspondence of the final WRF. 

The GH and GE membranes showed initial permeate fluxes of 57 kg/m^2^ h and 20 kg/m^2^ h, respectively; these values were reduced down to 37 kg/m^2^ h and 16 kg/m^2^ h, respectively, when the final WRF was reached. Higher fluxes for the GH membrane on the pilot scale in comparison to the GE membrane were also observed in the treatment of the aqueous extract of spent coffee at 5 bar and 40 °C [27]. 

The flux decline ratio (FDR) for the selected membranes followed the following order: GK > DK > GH > GE (50%, 47%, 35%, and 20%, respectively), and it was in agreement with the observed permeate flux values (Table 2). For the UF membranes, the FDR was in accordance with their MWCO—indeed, the GK membrane showed a higher FDR in comparison with the GH and GE membranes with a lower MWCO.

As expected, as the higher values of flux decline indicated more intense membrane fouling, the GK and the DK membranes showed the highest fouling index (34.03% and 27.05%, respectively), followed by the GE (22.03%) and GH (19.45%) membranes. For the UF membranes, we assume that the pore blocking gives a major contribution to the membrane fouling, which increases with the membrane pore size. This trend could be expected, as membranes with a higher MWCO are more sensitive to fouling [27]. 

The alkaline cleaning produced a good recovery of the initial water permeability, and the CE resulted in being higher than 91% for all of the tested membranes, following the following order: DK > GE > GH > GK. Sohrabi et al. [28] obtained similar results by studying the efficiency of the chemical cleaning in reverse osmosis and the NF membranes fouled by licorice aqueous solutions. The authors reported a flux recovery for both membranes of higher than 90% when using an NaOH solution at 0.1% for 5 min at 25 ± 1 °C. The increasing pH causes a negative charge of both organic molecules and membrane surface. An electrostatic repulsion mechanism is therefore considered as responsible for the high efficiency of the alkaline cleaning.

### 2.3. UF and NF of Clarified Licorice Wastewaters: Analyses of Membrane Selectivity

**Figure 4:**
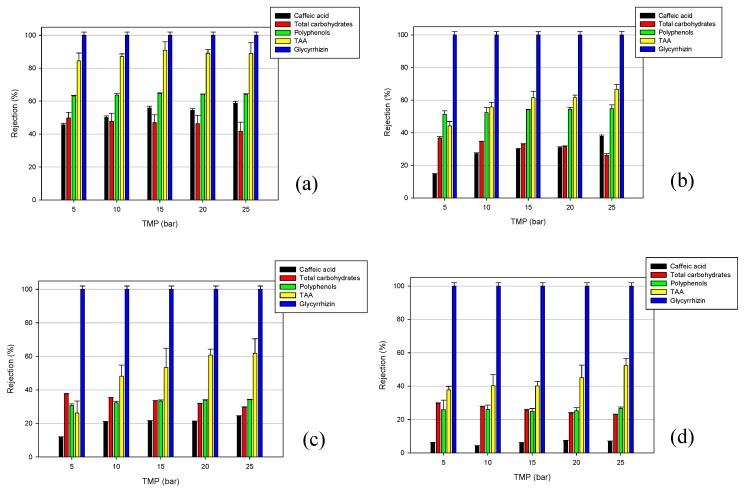
Rejection of total antioxidant activity (TAA) and solutes as a function of the transmembrane pressure (TMP) for the investigated membranes. (**a**) DK membrane; (**b**) GE membrane; (**c**) GH membrane; (**d**) GK membrane (T 23 ± 1 °C; Q_f_ 480 L/h).

The selected membranes were also compared in terms of their selectivity towards the analyzed compounds. Figure 4 shows the effect of TMP on the solute and TAA rejections for each tested membrane. The obtained results showed a complete rejection (100%) towards glycyrrhizic acid, independent of the MWCO of the membranes and the applied TMP. 

For the NF membrane (Figure 4a), this behavior could be attributed to the molecular weight (822.06 g/mol) of this component, which is higher than the MWCO of the NF membrane. High retention values of glycyrrhizin were also observed in the treatment of licorice root extract, with a composite polyamide membrane (NTR-7450) having an MWCO in the range of 600–700 Da [17]. In particular, the authors found an increase in rejection from 98.8% to 99.3%, by increasing the operating pressure from 4 to 10 bar. 

The high rejection level observed with the UF membranes (Figure 4b–d), despite their MWCO being higher than the molecular weight of glycyrrhizin, could be attributed to the charge effects. Glycyrrhizic acid is a weak acid with three carboxyl and five hydroxyl groups (pka1 = 2.76; pka2 = 2.81; pka3 = 4.71). At a pH of <3, most of the glycyrrhyzic acid exists in molecular form, while a dissociated form exhibiting a negative charge is predominant at higher pH values [29]. The pH of licorice wastewater is 4.82—at this pH, the surfaces of the selected UF membranes have a negative charge [30], leading to a retention of glycyrrhizin as a result of the electrostatic repulsion. This result confirms that for NF and tight UF membranes, the electrostatic interactions between the membrane surface and solutes can improve the membrane selectivity, if the chemical environment is favorable [31]. The association of glycyrrhizin with the retained macromolecules, as well as the steric effects, can also contribute to the observed rejection of glycyrrhizin [32]. 

A different trend was observed for the other analyzed compounds. In particular, from Figure 4, it is possible to note an increase in the rejection of the selected membranes towards caffeic acid, with the increasing of TMP and decreasing of MWCO (rejection values in the range of 46%–58%, 14%–37%, 11%–24%, and 6.2%–7% for the DK, GE, GH, and GK membranes, respectively). A similar trend was also observed for the TAA and total polyphenols. In particular, the rejection values for TAA were in the range 84%–89%, 44%–66%, 26%–62%, and 37%–52% for the DK, GE, GH, and GK membranes, respectively. On the other hand, the total polyphenols rejections ranged from 63% to 64% for the NF membranem and in the range of 51%–54%, 30%–34%, and 25%–27% for tight UF membranes (GE, GH, and GK, respectively).

These results are consistent with those of several studies that also reported increased retention rates of phenolic compounds by increasing the operating pressure [23,24,33,34]. This behavior is attributed to a more severe concentration of polarization and fouling phenomena at higher TMP values, leading to the formation of an additional selective layer on the membrane surface, and, therefore, an increase in the rejection coefficients. 

**Figure 5:**
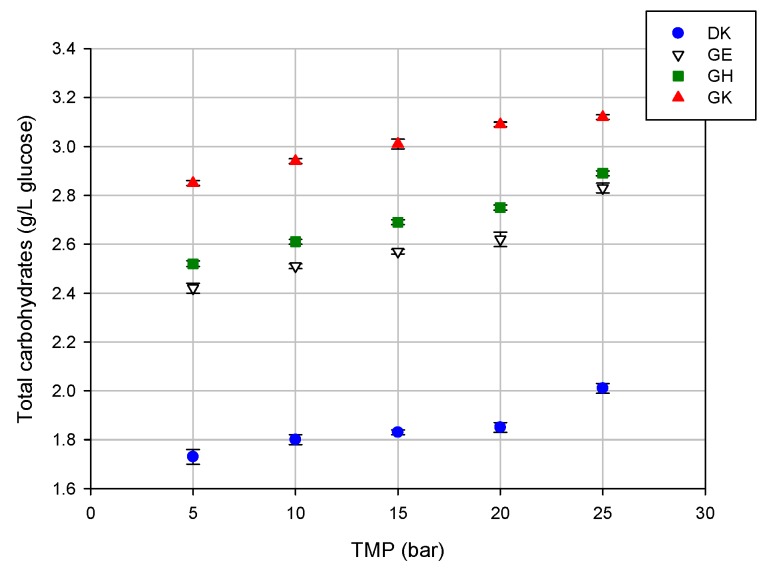
Concentration of total carbohydrates in the permeate fractions of the selected membranes at different TMP values (T 23 ± 1 °C; Q_f_ 480 L/h).

A different trend was observed for the total carbohydrates; indeed, for these compounds, the rejection values of the selected membranes decreased by increasing the applied TMP. In particular, the observed rejections were in the range of 50%–42%, 37%–26%, 38%–29%, and 30%–23% for the DK, GE, GH, and GK membranes, respectively. This result is due to the increased concentration of these compounds in the permeate stream by increasing the operating pressure (Figure 5).

**Figure 6:**
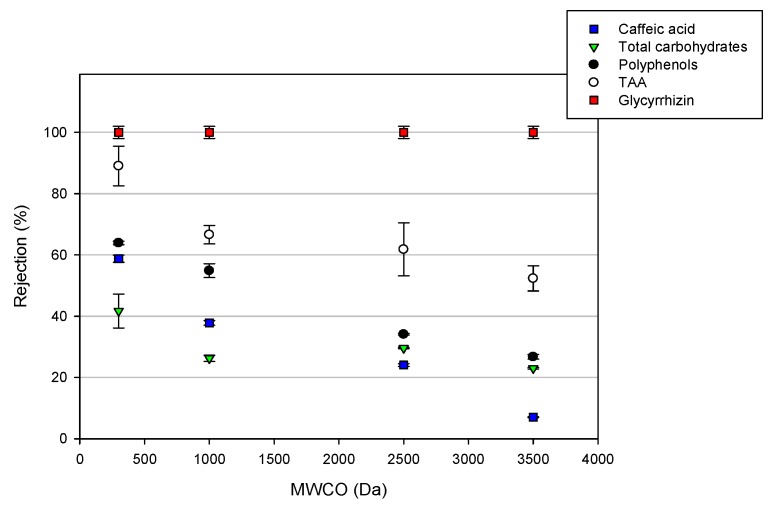
Effect of the nominal molecular weight cut-off (MWCO) on the rejection of UF and NF membranes towards the target compounds (TMP 25 bar; T 23 ± 1 °C; Q_f_ 480 L/h).

The total results show that the rejection values of the selected membranes towards the different analyzed compounds, with the exception of glycyrrhizic acid, are strongly dependent by the nominal MWCO, and decreased in the following order: DK > GE > GH > GK. At the operating pressure producing the maximum permeation flux (25 bar), the NF membrane with the lowest MWCO showed the highest rejection towards all of the analyzed compounds; on the other hand, the GK membrane with the highest MWCO showed increased separation factors between the glycyrrhizin and carbohydrates (Figure 6).

In Table 3, the composition of the feed, permeate, and retentate fractions collected for the selected membranes at different WRFs are reported. Glycyrrhizic acid was retained by all of the selected membranes—its concentration in the retentate fractions increased by increasing the WRF. On the other hand, the permeate fractions at different WRFs were completely depleted of this component. Similarly, the concentration of polyphenols in the retentate increased when the WRF was increased in the range of the investigated values. At WRF 5, the concentration of the phenolic compounds in the retentate fraction of the DK membrane was higher than that observed for the other selected membranes. 

An increase in the total polyphenols by increasing the volume reduction factor was also observed by Negrão Murakami et al. [35] in the concentration of an aqueous mate (*Ilex paraguariensis* A. St. Hil) extract through a spiral-wound NF membrane (HL2521TF form GE Osmonics, Minnetonka, USA), with an approximate MWCO of 150–300 Da. Similarly, Díaz-Reinoso et al. [36] reported an increased rejection of the phenolic compounds with the volume reduction factor in the treatment of *Castanea sativa* leave aqueous extracts with 5 and 10 kDa polyethersulfone membranes (Omega membranes, Pall Filtron, Northborough, MA, USA).

These results were in agreement with the measurement of TAA. In particular, the TAA decreased by increasing the WRF in the different permeate fractions; adversely, an increase of TAA in the retentate fractions by increasing the WRF was observed. As expected, the membranes with larger pores allowed for a higher recovery of antioxidant compounds in the permeate stream. At WRF 5, the TAA in the permeate fraction of the GK membrane was higher than that observed for the other tested membranes; on the other hand, the retentate fraction of the DK membrane at a WRF of 5 presented the highest antioxidant activity (21.1 ± 1.1 mM of trolox). This phenomenon can be attributed to the high concentration of total polyphenols, caffeic acid, and glycyrrhizin in this fraction. 

A similar trend was also observed for the caffeic acid and total carbohydrates. In this case, the concentration of caffeic acid in the permeate of the GK membrane at WRF 5 (22.05 ± 0.44 mg/L) was similar to that of the clarified solution (22.13 ± 0.44 mg/L), while for the total carbohydrates, the concentration in this fraction was about 25% lower than the feed solution. The lowest recovery of both components was recorded in the permeate stream of the DK membrane, in accordance with the lowest MWCO.

### 2.4. Integrated Membrane Process 

**Figure 7:**
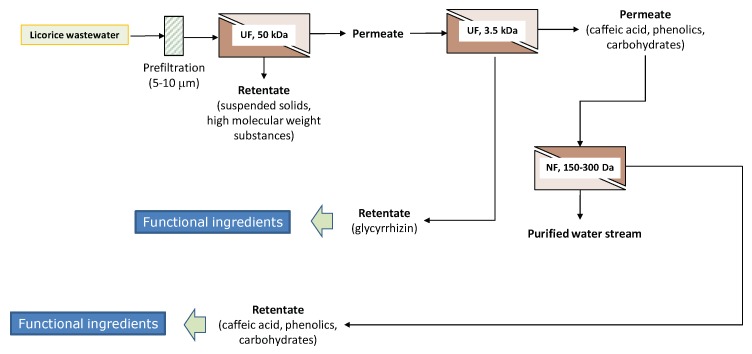
Conceptual process design for the treatment of licorice aqueous wastewaters with UF and NF membranes.

On the basis of the experimental results, an environmentally-friendly integrated process for the separation and purification of glychirrizic acid and other biologically active compounds from licorice wastewaters has been proposed (Figure 7). Raw wastewaters were previously clarified by UF in order to remove suspended solids, colloidal substances, and high molecular weight substances, with the production of a clear permeate enriched of glycyrrhizic acid and other biologically active compounds. The pretreated solution is processed through a thin-film composite membrane with a MWCO of 3500 Da, in order to separate the glycyrrhizic acid from the caffeic acid, phenolic compounds, and carbohydrates—the produced retentate stream is enriched by a fraction in the glycyrrhizic acid of interest as a functional ingredient; the permeate stream is concentrated by a thin-film composite membrane with a MWCO of 150–300 Da, producing a water stream (permeate fraction) that can be reused as processing water, and a retentate stream enriched in the biologically active compounds of interest for the production of functional formulations with a high antioxidant capacity.

## 3. Materials and Methods 

### 3.1. Licorice Aqueous Solutions 

Licorice wastewaters were provided by Gioia Succhi S.R.L. (Rosarno, Reggio Calabria, Italy). They were stored at −17 °C and were defrosted at room temperature before use. Before the clarification process, the aqueous solutions were filtered through a cotton fabric filter.

### 3.2. Clarification of Licorice Wastewater 

Licorice wastewaters were submitted to a preliminary clarification process using a laboratory unit supplied by Verind SpA (Milan, Italy) equipped with a polysulphone hollow fiber membrane module (DCQ III-006), supplied by China Blue Star Membrane Technology (Beijing, China), with a molecular weight cut-off (MWCO) of 50 kDa and a membrane surface area of 1.2 m^2^. The UF was operated at a transmembrane pressure (TMP) of 0.26 bar, an axial feed flowrate (Q_f_) of 516 L/h, and a temperature (T) of 24 ± 1 °C, according to a batch concentration configuration (recycling the retentate stream in the feed tank and collecting the permeate separately), up to a weight reduction factor (WRF; defined as the ratio between the initial feed weight and the weight of the resulting retentate) of 7.5. A cleaning-in-place procedure was used in order to recover the original water permeability of the UF membranes after licorice wastewater clarification. In particular, the UF membranes were firstly washed with water to remove the unbounded substances from the membrane surface, and then submitted to a chemical cleaning with an alkaline solution (Ultraclean WA at 0.2% *w*/*w*). The solution was recycled through the lumen side of the membrane module at a TMP of 0.10 bar, a Q_f_ of 560 L/h, and an average temperature of 40 °C for 60 min. Finally, the circuit was rinsed with tap water. 

### 3.3. Fractionation of Clarified Solution with UF and NF Membranes: Set-Up and Procedures

The clarified liquor was subjected to a fractionation/concentration step performed by UF or NF. Both of the processes were carried out using a pilot unit manufactured by DeltaE s.r.l. (Cosenza, Italy), equipped with a stainless-steel housing able to accommodate a spiral-wound membrane module featuring an effective membrane area of 0.32 m^2^. The equipment consists of a feed tank with a capacity of 5 L, a high-pressure pump, a digital flowmeter, and a control panel. The feed temperature was adjusted by circulating tap water in the two-layered feed tank. The pressure was monitored at the entrance and exit of the membrane module; it was controlled by a back-pressure control valve located after the membrane module, and by setting the pump speed on the control panel. 

The experiments were performed using four different spiral-wound membrane modules manufactured by GE Osmonics and purchased from Sepra S.r.l. (Cesano Maderno, Italy). Their properties are reported in Table 4. 

The experimental trials were performed in full recycle mode (the feed composition was kept constant by recycling both permeate and retentate to the feed tank), in order to evaluate the influence of TMP on the permeate flux and the rejection of membranes towards target compounds. The TMP values were modified in the range 5–25 bar, at fixed conditions of T (23 ± 1 °C) and Q_f_ (480 L/h). A constant feed quantity of clarified solution (3.5 kg) was used for each experiment. 

Batch concentration experiments were also performed in selected operating conditions (TMP 25 bar; Q_f_ 480 L/h; T 23 ± 1 °C), up to a WRF of 5, in order to evaluate the effect of WRF on the permeate flux and selectivity. 

The membrane performance was evaluated as permeate flux (*J_p_*), fouling index (*FI*), cleaning efficiency (*CE*) and selectivity towards total polyphenols, caffeic acid, total carbohydrates, and glycyrrhizic acid.

The permeate flux was monitored by measuring the permeate weight collected at fixed time intervals using a digital balance according to the following equation:

(1) $$J_{p}=\frac{W_{p}}{A\;t}$$

where *J_p_* is the permeate flux (kg/m^2^ h), *W_p_* the permeate weight (kg) at time *t* (h), and *A* the membrane surface area (m^2^).

The flux decline ratio (*FDR*) was calculated as the ratio of the initial permeate flux to the final permeate flux measured at the end of the experiments. 

The fouling index (*FI*) of the selected membranes, expressed as the percentage drop in water permeability, was estimated by measuring the water permeability before and after the treatment of clarified licorice root extract, according to the following equation:

(2) $$FI=\left(\frac{W_{p1}}{W_{po}}\right)\cdot 100$$

where *W_p0_* and *W_p1_* are the pure water permeability before and after licorice wastewater filtration, respectively. 

The water permeability of each membrane was determined by the slope of the straight line obtained by plotting the water flux values, measured in fixed conditions of T (25 ± 1 °C), versus the applied TMP. 

The fouled membranes were cleaned with an alkaline detergent (0.2% Ultraclean WA, from Henkel Ecolab), at 40 ± 1 °C for 40 min in full recycle mode. After that, the system was rinsed with water in order to assess the cleaning efficiency (*CE*), according to the following equation:

(3) $$CE=\left(\frac{W_{p2}}{W_{p0}}\right)\cdot 100$$

where *W_p2_* is the pure water permeability measured after the chemical cleaning. 

The feed, permeate, and retentate samples collected during the full recycle and batch concentration experiments at different TMP and WRF, respectively, were kept in a freezer (−20 °C) up until the analysis. 

The rejection (*R*) of the UF and NF membranes towards specific compounds, depending on whether evaluated in total recycle or batch concentration mode, was calculated according the following equations, respectively:

(4) $$R=\left(1-\frac{c_{p}}{c_{f}}\right)\cdot 100$$

(5) $$R=\left(1-\frac{c_{p}}{c_{r}}\right)\cdot 100$$

where *c_p_*, *c_f_*, and *c_r_* are the permeate, feed, and retentate solute concentrations, respectively.

### 3.4. Analytical Measurements

The feed (F), permeate (P) and retentate (R) samples coming from the full recycle and batch concentration experiments were analyzed to quantify the suspended solids (SSs), total polyphenols, total antioxidant activity (TAA), total carbohydrates, glicyrrhizic acid, and caffeic acid. The results of the analytical measurements were expressed as mean ± standard deviation (SD) of three independent determinations.

#### 3.4.1. Determination of Suspended Solids

The SSs were determined by centrifuging 10 mL of a pre-weight sample at 2000 rpm for 20 min; the weight of settled solids was determined after removing the supernatant.

#### 3.4.2. Determination of Total Polyphenols

The total polyphenols were estimated colorimetrically using the Folin–Ciocalteu method [39]. The method is based on the reduction of tungstate and/or molybdate in the Folin–Ciocalteu reagent by phenols in an alkaline medium, resulting in a blue colored product. Gallic acid was used as a calibration standard and the results were expressed as the gallic acid equivalent (mgGAE/L). The absorbance was measured using a UV-visible spectrophotometer (ShimadzuUV-160A, Kyoto, Japan) at 765 nm.

#### 3.4.3. Determination of Glicyrrhizic Acid and Caffeic Acid

Glicyrrhizic acid and caffeic acid were analyzed using an HPLC system equipped with an UV detector (Agilent 1200 Series, Sanya Clara, CA, USA) and a Luna C 18(2) column (250 × 4.6 mm, 5 µm Phenomenex, Torrance, CA, USA); the following conditions were used: V = 1 mL min; T = 25 °C; λ = 254 nm. The mobile phase was a mixture of H_2_O/HCOOH (9:1) as solvent A, and CH3CN as solvent B. 

The following gradient system was used: starting condition 80% and 20% B; 12 min, 60% A and 40% B; 20 min, 80% A and 20% B; The analyses were stopped after 24 min. Prior to the HPLC analysis, all of the samples were filtered using 0.45 mm nylon filters. Glicyrrhizic acid and caffeic acid acids were identified by matching the retention time and their spectral characteristics against those of the standards. Quantization was made according to the linear calibration curves of the standard compounds. Each assay was performed in triplicate. The deviation of each measurement was of 2% from the average value.

#### 3.4.4. Determination of Total Antioxidant Activity

The total antioxidant activity (TAA) was determined according to an improved version of the 2,2-azino-bis- (3-ethylbenzothiazoline-6-sulphonic acid (ABTS) radical cation decolourisation assay, in which the radical monocation (ABTS^+^) is generated by the oxidation of ABTS (Sigma Aldrich, Milano, Italy) with potassium persulphate (Sigma Aldrich, Milano, Italy), before the addition of the antioxidant [40]. The results were expressed as trolox equivalent antioxidant capacity (TEAC).

#### 3.4.5. Determination of Total Carbohydrates

The total carbohydrates were measured using the phenol–sulfuric acid method [41]. A sample aliquot (0. 2 mL) of a carbohydrate solution was mixed with 1 mL of 5% aqueous solution of phenol in a test tube. Then, 5 mL of concentrated sulfuric acid was added rapidly to the mixture. After allowing the test tubes to stand for 10 min, they were vortexes for 30s and placed for 30 min in a water bath at room temperature for color development. The absorbance was measured at 420 min using an UV-visible spectrophotometer (Shimadzu UV-160 A, Kyoto, Japan). Glucose solutions with concentrations ranging from 0.02 e 0.1 g/L were used for the calibration. A dose response linear regression was generated using the glucose standard absorbance, and results were expressed as g/L of glucose.

## 4. Conclusions

The potential of the commercial spiral-wound UF and NF membranes for the recovery of high-added value compounds from licorice wastewaters was evaluated. Among the investigated membranes, the UF membrane with a MWCO of 3500 Da had proven to be the most appropriate membrane in the treatment of clarified wastewaters exhibiting the highest productivity, as well as the highest separation capability of glycyrrhizic acid from other biologically active compounds (in selected operating conditions, more than 83% of the glycyrrhizic acid was recovered in the final retentate, while about 80% of the caffeic acid was recovered in the permeate stream). On the other hand, the NF membrane with a MWCO of 50–300 Da assured the rejection of other desirable bioactive compounds and carbohydrates from clarified wastewaters. Therefore, the combination of these membranes in a sequential design is proposed in order to fractionate and refine antioxidant-containing solutions from the licorice industry. The proposed flow sheet allows for producing two different concentrated fractions enriched in bioactive compounds, mainly glycyrrhizin and phenolic compounds, respectively, of interest for the formulation of functional foods, nutraceuticals, and cosmeceuticals. A purified water stream that can be reused in the working cycle or disposed in water bodies is also produced within the logic of a zero discharge approach.

## Figures and Tables

**Table 1:** Chemical composition of licorice wastewaters before and after the clarification with the UF membrane. TAA—total antioxidant activity; n.d.—not detectable

Parameter	Feed	Permeate	Retentate
Suspended solids (%, *w*/*w*)	1.9 ± 0.1	n.d.	5.6 ± 0.2
Total carbohydrates (g/L)	5.03 ± 0.01	4.24 ± 0.03	8.11 ± 0.01
Glycyrrhizic acid (mg/L)	224.3 ± 4.5	210.1 ± 4.20	256.35 ± 5.13
Caffeic acid (mg/L)	27.1 ± 0.5	24.3 ± 0.5	28.1 ± 0.6
TAA (mM Trolox)	6.0 ± 0.6	5.9 ± 2.8	6.2 ± 0.7
Total polyphenols (mg/L gallic acid)	883.7 ± 30.7	844.9 ± 26.2	1165.9 ± 8.8

**Table 2:** Flux decline ratio, index of fouling, and cleaning efficiency of the selected membranes. FDR—flux decline ratio; FI—fouling index; CE—cleaning efficiency.

	Membrane Type			
	GK	GH	GE	DK
FDR (%)	50	35	20	47
FI (%)	34.03	19.45	22.03	27.25
CE (%)	91.50	92	98.23	100

**Table 3:** Physicochemical characteristics of the clarified licorice extracts submitted to the UF and NF treatments. WRF—weight reduction factor; n.d.—not detectable)

Membrane Type	Sample	WRF	Total Polyphenols (mg/L)	Total Carbohydrates (mg/L)	Caffeic Acid (mg/L)	Glycyrrhizin (mg/L)	TAA (mM Trolox)
**GK**	Feed		764.5 ± 12.3	3.99 ± 0.03	22.1 ± 0.4	207.3 ± 4.1	5.1 ± 0.4
	Permeate	2	604.6 ± 6.1	2.86 ± 0.03	21.8 ± 0.4	n.d.	3.7 ± 0.4
		3	630.6 ± 37.0	2.89 ± 0.01	22.1 ± 0.4	n.d.	3.6 ± 0.5
		4	595.9 ± 20.5	2.93 ± 0.01	22.1 ± 0.4	n.d.	3.4 ± 0.3
		5	587.2 ± 5.8	2.99 ± 0.02	22.1 ± 0.4	n.d.	3.3 ± 0.1
	Retentate	2	1017.4 ± 32.9	4.41 ± 0.02	27.1 ± 0.5	377.1 ± 7.5	7.4 ± 0.2
		3	1186.1 ± 49.3	5.53 ± 0.02	35.1 ± 0.7	578.3 ± 11.5	8.4 ± 0.3
		4	1337.2 ± 8.2	7.32 ± 0.02	42.3 ± 0.8	755.7 ± 15.1	9.1 ± 0.5
		5	1395.3 ± 8.2	8.10 ± 0.01	54.1 ± 1.1	863.6 ± 17.3	10.5 ± 0.4
**GH**	Feed		746.1 ± 3.3	4.12 ± 0.03	20.5 ± 0.4	192.5 ± 3.8	5.0 ± 0.5
	Permeate	2	523.2 ± 5.8	2.40 ± 0.01	19.5 ± 0.2	n.d.	3.6 ± 0.4
		3	534.8 ± 5.8	2.43 ± 0.01	20.1 ± 0.4	n.d.	3.3 ± 0.1
		4	536.8 ± 6.7	2.52 ± 0.01	19.8 ± 0.4	n.d.	3.0 ± 0.4
		5	529.1 ± 5.8	2.61 ± 0.02	20.1 ± 0.4	n.d.	2.8 ± 0.3
	Retentate	2	1127.9 ± 5.8	4.49 ± 0.04	35.6 ± 0.7	282.0 ± 5.6	6.9 ± 0.9
		3	1246.1 ± 12.1	5.70 ± 0.03	50.8 ± 1.0	412.0 ± 8.2	8.0 ± 0.7
		4	1492.2 ± 12.1	8.11 ± 0.01	59.4 ± 1.2	768.0 ± 15.3	10.0 ± 0.7
		5	1908.9 ± 8.8	9.05 ± 0.06	89.1 ± 1.8	900.6 ± 18.0	13.6 ± 0.5
**GE**	Feed		775.2 ± 3.3	4.10 ± 0.05	20.7 ± 0.4	173.9 ± 3.5	5.3 ± 0.4
	Permeate	2	434.1 ± 3.3	2.08 ± 0.01	18.1 ± 0.3	n.d.	3.0 ± 0.3
		3	395.3 ± 10.1	2.10 ± 0.01	17.1 ± 0.3	n.d.	2.6 ± 0.3
		4	374.0 ± 6.7	2.14 ± 0.02	17.3 ± 0.3	n.d.	2.3 ± 0.5
		5	407.0 ± 4.1	2.17 ± 0.02	18.3 ± 0.3	n.d.	2.6 ± 0.4
	Retentate	2	1129.8 ± 23.5	5.30 ± 0.03	36.1 ± 0.7	316.7 ± 6.3	7.4 ± 0.6
		3	1455.4 ± 16.8	6.73 ± 0.03	47.5 ± 0.9	485.1 ± 9.7	9.3 ± 3.3
		4	1839.1 ± 18.7	8.61 ± 0.46	79.9 ± 1.6	642.9 ± 12.9	9.6 ± 0.1
		5	2071.7 ± 13.4	9.21 ± 0.06	94.5 ± 1.9	792.1 ± 15.8	12.8 ± 0.8
**DK**	Feed		799.4 ± 20.5	4.08 ± 0.02	19.3 ± 0.4	208.2 ± 4.2	4.9 ± 0.3
	Permeate	2	319.7 ± 8.2	1.64 ± 0.02	11.4 ± 0.2	n.d.	1.7 ± 0.4
		3	308.1 ± 3.1	1.64 ± 0.03	12.3 ± 0.2	n.d.	1.5 ± 0.4
		4	305.2 ± 4.1	1.73 ± 0.02	13.1 ± 0.2	n.d.	1.5 ± 0.5
		5	293.6 ± 4.1	1.75 ± 0.02	12.4 ± 0.2	n.d.	0.7 ± 0.5
	Retentate	2	1180.2 ± 10.1	5.86 ± 0.01	32.1 ± 0.6	315.9 ± 6.3	9.6 ± 0.2
		3	1363.4 ± 12.3	7.55 ± 0.01	43.5 ± 0.8	534.9 ± 10.7	11.6 ± 0.3
		4	1857.5 ± 20.5	9.13 ± 0.06	52.8 ± 1.1	823.9 ± 16.5	15.6 ± 0.9
		5	2247.1 ± 20.5	10.11 ± 0.06	90.1 ± 1.8	1085.8 ± 21.7	21.2 ± 1.1

**Table 4:** Characteristics of the selected spiral-wound membranes. PA-TFC—polyamide thin-film composite; MWCO—molecular weight cut-off.

Membrane Type	DK	GE	GH	GK
Manufacturer	GE Osmonics	GE Osmonics	GE Osmonics	GE Osmonics
Membrane material	PA-TFC	PA-TFC	PA-TFC	PA-TFC
Nominal MWCO (Da)	150–300	1000	2500	3500
pH operating range	3–9	2–10	2–10	2–10
pH range in cleaning conditions	2–10.5	1–11.5	1–11.5	1–11.5
Max. operating temperature (°C)	50	50	50	50
Max. operating pressure (bar)	41	27.6	27.6	27.6
Membrane surface area (m^2^)	0.32	0.32	0.32	0.32
Contact angle (°)	37.9 ± 3.3 ^a^	73.0 ± 0.5 ^b^	62.0 ± 1.1 ^b^	71.0 ± 1.4 ^b^
Average pore width (nm)	9.6 ^a^	-	-	-
Mean pore diameter (nm)	-	1.83 ± 0.35 ^c^	2.23 ± 0.46 ^c^	2.52 ± 0.51 ^c^

^a^ data from Vieira et al. [37]; ^b^ data from Tres et al. [30]; ^c^ data from Bowen et al. [38].
